# Study on Welding Mechanism Based on Modification of Polypropylene for Improving the Laser Transmission Weldability to PA66

**DOI:** 10.3390/ma8084961

**Published:** 2015-08-04

**Authors:** Huixia Liu, Hairong Jiang, Dehui Guo, Guochun Chen, Zhang Yan, Pin Li, Hejun Zhu, Jun Chen, Xiao Wang

**Affiliations:** 1 School of Mechanical Engineering, Jiangsu University, Xuefu Road, Zhenjiang 212013, China; E-Mails: 15751010042@163.com (H.J.); nylgxyguodehui@163.com (D.G.); chgcujs@163.com (G.C.); yanzhang1233@163.com (Z.Y.); lipin66888@163.com (P.L.); wx@ujs.edu.cn (X.W.); 2 School of Mechanical and Electrical Engineering, Zhenjiang Vocational Technical College, Xuefu Road, Zhenjiang 212013, China; E-Mails: hehe666777@163.com (H.Z.); cj1969@163.com (J.C.)

**Keywords:** laser transmission welding (LTW), the graft modified polypropylene (TGMPP), PA66, maleic anhydride (MAH)

## Abstract

Polypropylene and PA66 are widely used in our daily life, but they cannot be welded by laser transmission welding (LTW) because of polar differences and poor compatibility. In this paper, grafting modification technology is used to improve the welding performance between polypropylene and PA66. Firstly, the strong reactive and polar maleic-anhydride (MAH) is grafted to polypropylene and infrared spectrometer is used to prove that MAH has been grafted to polypropylene. At the same time, the mechanical and thermal properties of the graft modified polypropylene (TGMPP) are tested. The results prove that the grafting modification has little influence on them. Also, the optical properties of TGMPP are measured. Then, the high welding strength between TGMPP and PA66 is found and the mechanism of the weldability is researched, which shows that there are two reasons for the high welding strength. By observing the micro morphology of the welding zone, one reason found is that the modification of polypropylene can improve the compatibility between polypropylene and PA66 and make them easy to diffuse mutually, which causes many locking structures formed in the welding region. The other reason is that there are chemical reactions between TGMPP and PA66 proved by the X-ray photoelectron spectrometer.

## 1. Introduction

In the field of microelectronics, biomedicine and automobile parts, many products are made of polymers, which make the connection of polymers a significant problem [1]. Through the connection of dissimilar polymers, excellent performances of different polymers can be combined. But different from the connection between the same kinds of polymers, the connection of dissimilar polymers has many new challenges because of different chemical and thermal properties. Traditional methods of connecting dissimilar polymers are mechanical interlock, mechanical connection (screws/nuts and bolts/rivets), adhesive, fusion bonding, hot plate welding, brazing process and so on. Ageorges *et al.* looked at the state of the art of fusion bonding technology and reviewed the application of fusion bonding to joining dissimilar materials [2]. Qiu *et al.* investigated the effects of welding conditions on the interfacial microscopic structure and welding strength of hot plate welded polypropylene [3]. Obviously, these traditional methods may play an important role in different applications. However, these methods have some obvious defects, such as bad welding quality, the introduction of impurities and so on. Liu *et al.* used response surface methodology to develop mathematical models between the key process parameters and the desired responses and utilized the central composite design to conduct experimental planning when they welded polyethylene terephthalate (PET) and titanium coated glass by LTW [4]. Wang *et al.* investigated an intelligent method for simulation and optimization of continuous laser transmission welding and validated it with experiments in LTW of PET and titanium [5]. Acherjee *et al.* made a systematic investigation of the laser transmission contour welding process by using finite element analysis and design of experiments techniques in LTW of natural polycarbonates (PC) and opaque PC [6]. It can be found that compared to the traditional connecting methods, LTW has many advantages, such as precision, strong and sealing weld seam, ease of control, ability to realize automatic production, high welding speed, high quality, small welding thermal stress and vibration stress and so on [4,5,6]. Therefore, LTW is widely used in many sectors like microelectronics, biomedicine and automotive industries [7,8].

There have been many researches on LTW of dissimilar polymers recently. Wang *et al.* made a systematical investigation into the relationships of process parameters, molten pool geometry and shear strength in LTW of PET and polypropylene (PP) [9]. Acherjee *et al.* connected polymethylmethacrylate (PMMA) and acrylonitrile-butadiene-styrene copolymer (ABS) by LTW, and studied the influence of welding parameters on welding strength and weld seam width [10]. At present, the welding of dissimilar polymers which can be welded originally is the main object of researches. However, there are only few researches to improve the laser transmission welding performance of dissimilar polymers which cannot be welded together at first because of large polar and melting point difference. As commonly used plastics, PP and PA66 have wide application and are highly consumed. O’Connor *et al.* proposed that PP has small water absorption, is lightweight, easy to process and low cost [11]. The characters of PA66 are good elasticity, wear resistance, good self-lubricating properties, oil resistance, good chemical stability and the advantage of excellent processing fluidity [12,13]. If PP and PA66 can be welded together by laser, their respective advantages will be fully utilized and they will have great application prospects. PP is a kind of non-polar polymer while PA66 is polar, which makes them difficult to be welded together. Given this situation, TGMPP is researched to make the welding performance of PP and PA66 better with MAH grafted to the side chain of PP to improve the compatibility and affinity between them. Also, the laser transmission welding performance between TGMPP and PA66 and the welding mechanism based on modification of PP for improving the laser transmission weldability to PA66 are studied.

## 2. The Graft Modified Polypropylene

### 2.1. The Preparation of TGMPP and Its Infrared Spectroscopy

**Figure 1:**
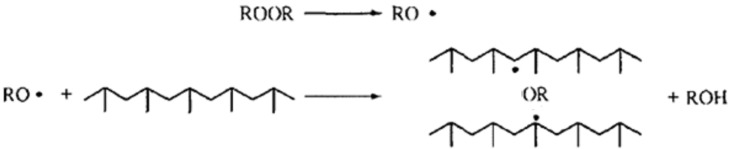
The formation of polypropylene macromolecule free radical.

**Figure 2:**
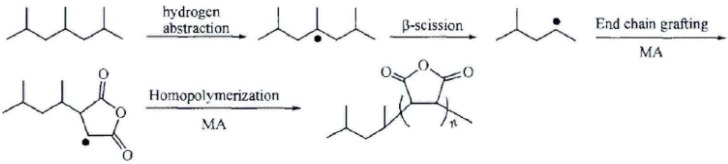
Polypropylene grafting mechanism (1).

**Figure 3:**
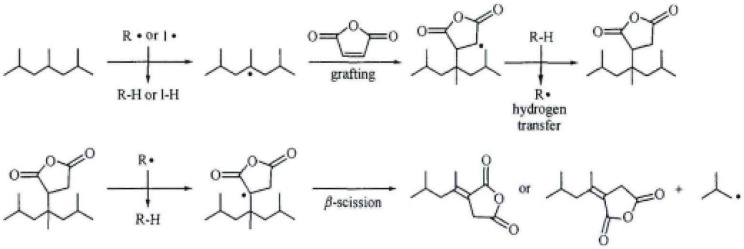
Polypropylene grafting mechanism (2).

Mixed at the ratio of 4:0.3, MAH and dicumyl peroxide (DCP) are dissolved by acetone. Then, the mixed solution is poured into 100 parts of PP, which is fully mixed by the high speed mixing machine at 5 min. At last, the mixture is put into a co-rotating twin-screw granulator unit (SHJ-35) to graft. The parameters are that the process temperature of extruder is Region I: 155 °C, II: 165 °C, III: 185 °C, IV: 190 °C, V: 195 °C, VI: 190 °C, VII: 180 °C,VIII: 175 °C and 170 °C at the head, the feeding speed is 10 r/min and the double screw speed is 30 r/min. The grafting modification is free radical reactions. Firstly, the initiator DCP seizes hydrogen on polypropylene tertiary carbon to form polypropylene macromolecule free radicals as shown in Figure 1. Then, the competitive reactions which are (1) grafted with MAH or (2) the rupture of beta bond will happen. When the concentration of MAH is low, most of MAH is grafted to the end of the active macromolecules produced by the rupture of beta bond as shown in Figure 2 while the MAH grafted to the polypropylene tertiary carbon free radicals is more than grafted to the end of the active macromolecules when the concentration of MAH is high as shown in Figure 3.

To improve the welding performance of PP and PA66, the strong polar and reactive MAH is grafted to PP with the objective of improving their compatibility and affinity.

Crushed and wrapped with filter paper, TGMPP is put into the Soxhlet apparatus using ethanol as a solvent and extracted about 24 h in a heating circumfluence water bath. Afterward, the products are dried for about 12 h in a 110 °C vacuum oven.

**Figure 4:**
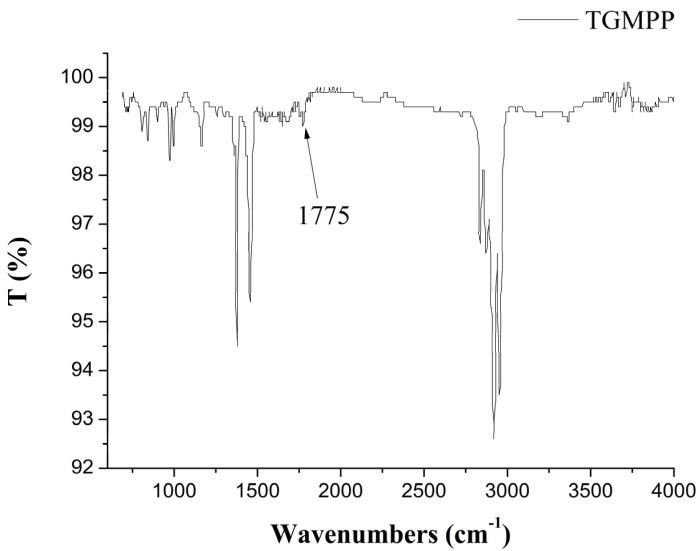
The infrared spectroscopy of TGMPP.

Mixed with pure KBr powder, the trace purified graft modified polypropylene is ground to solid powder. Then, the solid power is pressed into a transparent sheet, which is put into the optical path of the infrared spectrometer and scanned from 690 cm^−1^ to 4000 cm^−1^ at 40 times per minute. The infrared spectroscopy of TGMPP is shown in Figure 4. Similar to the infrared spectroscopy of PP, this spectrum has the bending vibration spectrum of CH_2_ at 1457 cm^−1^, the bending vibration spectrum of methyl at 1377 cm^−1^, multiple peaks at 2800~3000 cm^−1^ and the characteristic peaks of [CH_2_CH(CH_3_)]_m_ at 973 cm^−1^ and 1166 cm^−1^. However, there is a characteristic peak at 1775 cm^−1^ which cannot be found in the infrared spectroscopy of PP. Belonging to the peak of the MAH which is grafted to PP, the characteristic peak proves that the MAH has been grafted to the molecular chain of PP.

### 2.2. The Mechanical and Thermal Properties of TGMPP

**Figure 5:**
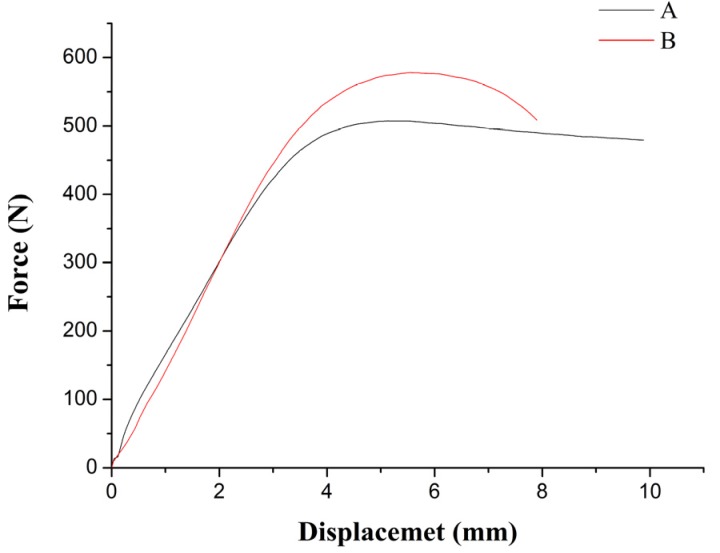
The force-displacement curve diagram of PP and TGMPP.

To do the tensile mechanical experiments which are used to explore the tensile mechanical properties of TGMPP, PP and TGMPP are made into L50 mm × W20 mm × H1 mm. The tensile speed is 2 mm/min and the working environment is room temperature when the tensile mechanical experiments are carried out. The tensile results are shown in Figure 5. In the figure, the A curve is the force-displacement diagram of PP and the B curve is the force-displacement diagram of TGMPP. It is not difficult to find that the ultimate strength of TGMPP is higher than PP while elongation is lower which confirms that the grafting modification reduces the plasticity of PP. Furthermore, the impacting property of TGMPP is a little lower than PP in the impacting tests.

**Figure 6:**
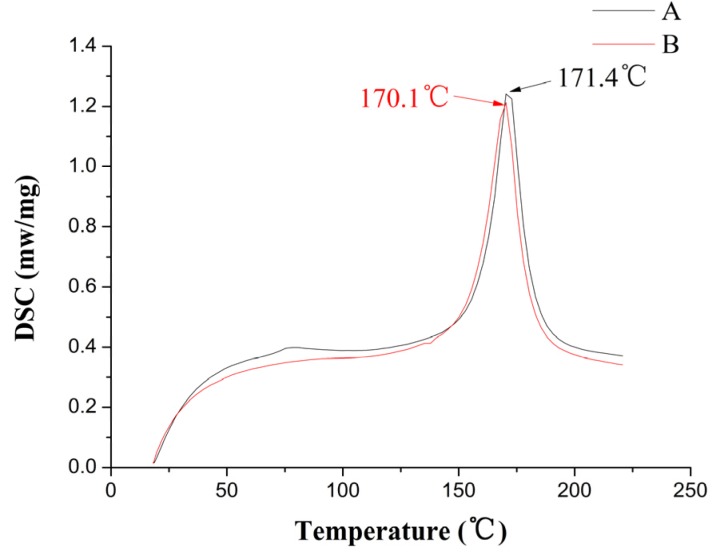
The DSC curves of PP and TGMPP.

Taking PP and TGMPP 10 mg, respectively, a dynamic heat flux differential scanning calorimeter is used to measure their melting peaks. The results are shown in Figure 6. In the figure, the A curve is the melting curve of PP and the B curve is the melting curve of TGMPP. It is easy to find that the grafting modification makes the melting point of PP slightly down about 1.3 °C (the melting point of PP is 171.4 °C and TGMPP is 170.1 °C), whose reason is the loss of molecular weight and the narrowing molecular weight distribution caused by the rupture of the molecular chain of PP under the action of initiator (DCP) in the reactive extrusion process.

In general, the grafting modification has a certain influence on the mechanical and thermal properties of PP. However, the original properties of PP change only a little.

### 2.3. The Effect of Grafting Modification on the Optical Properties of Polypropylene

The optical properties of polymers have great influence on the welding quality, so research of the effect of grafting modification on the optical properties of PP is very important. The optical properties include transmissivity, reflectivity and absorptivity. For all of laser transmission welding, it is hoped that the laser energy can transmit to the interface of the upper materials and the lower materials to improve the utilization efficiency of laser energy and reduce the processing cost.

Due to the loss of the laser energy, the laser energy density will decrease. Some of the laser energy loss is due to the reflection of the upper surface and the lower surface of the upper materials and the reflection of the interface of different solid components (additives, impurities and so on) in the upper materials. The reflectivity (*RT*) is defined as the ratio of the reflected energy and the total laser energy input.

When the laser penetrates the upper materials, some laser energy is absorbed by the impurities and the upper materials. Similar to the definition of reflectivity, the absorptivity (*AT*) is defined as the ratio of the absorbed energy and the total laser energy input. Then, the remaining laser energy passes through the upper materials to the interface of the upper materials and the lower materials. The transmissivity (*TT*) is defined as the ratio of this remaining laser energy and the total laser energy input. The reflectivity, absorptivity and transmissivity of one kind of material meet the following formula:

(1) RT +AT +TT=1

The reflectivity, absorptivity and transmissivity of TGMPP are researched in the next experiments. The transmissivity and reflectivity of TGMPP are measured by the ultraviolet-visible-near infrared absorption spectroscopy. The absorptivity of TGMPP is calculated based on Equation (1).

#### 2.3.1. The Effect of Grafting Modification on the Reflectivity of Polypropylene

**Figure 7:**
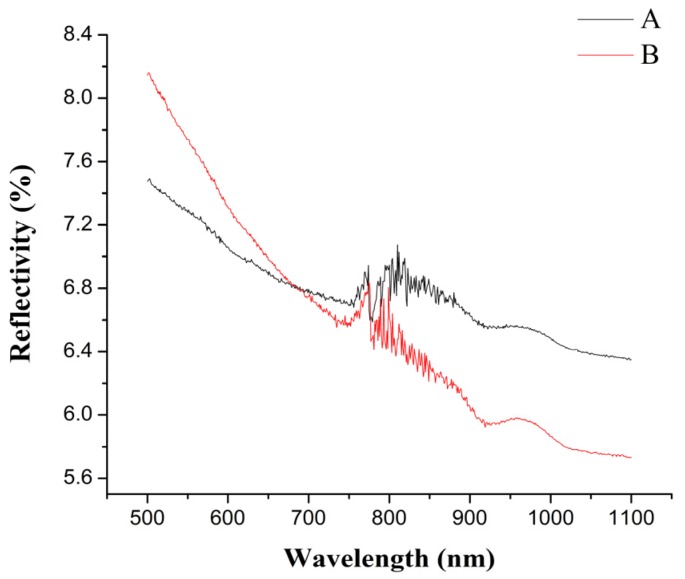
The reflectivity of PP at different wavelengths before and after grafting modification.

If the polymers are the upper materials, the laser reflection of the upper materials will transmit part of the laser energy back into the air, which results in a waste of energy and is bad for cost savings. Furthermore, if the laser reflectivity of the upper materials is too high, these upper materials cannot be welded with the lower materials by laser. So, the research of the reflectivity of TGMPP is very important. The reflectivity of PP at different wavelengths before and after grafting modification are shown in Figure 7, in which A curve represents the reflectivity of PP at different wavelengths before grafting modification (PP) and B curve represents the reflectivity of PP at different wavelengths after grafting modification (TGMPP). It can be seen from the figure that when the wavelength is 980 ± 10 nm (the laser wavelength in the next experiments), the grafting modification causes the reflectivity of PP to decrease slightly and reduces the loss of laser energy (TGMPP and PP are the upper materials in the next laser transmission welding experiments), which is good for the laser transmission welding.

#### 2.3.2. The Effect of Grafting Modification on the Transmissivity of Polypropylene

**Figure 8:**
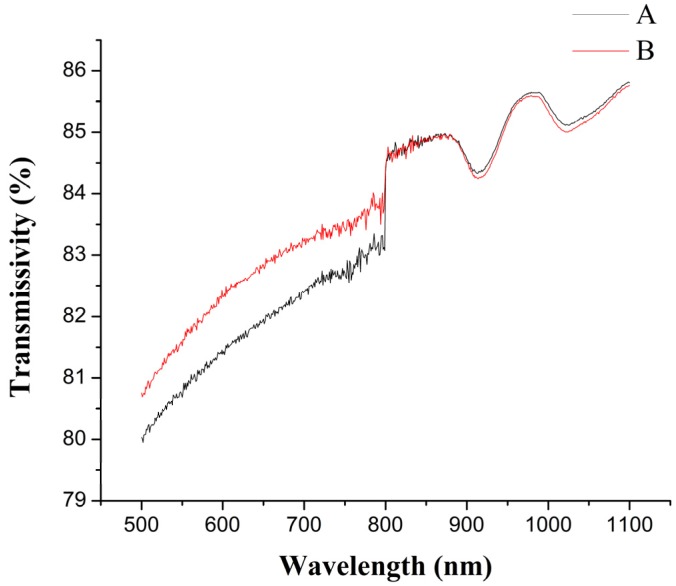
The transmissivity of PP at different wavelengths before and after grafting modification.

If the polymers are the upper materials, the high transmissivity of the upper materials will cause more laser energy to transmit to the welding zone while the low transmissivity of the upper materials will cause only a little laser energy to transmit to the welding zone, which is bad for cost savings. Furthermore, if the transmissivity of the upper materials is too low, it will prevent them from being welded with the lower materials under some extreme conditions. If the polymers are the lower materials and the transmissivity of the lower materials is high, most of the laser energy will pass through the lower materials to the air, which is also bad for the laser transmission welding. So, the research of the transmissivity of TGMPP is very important. The transmissivity of PP at different wavelengths before and after grafting modification are shown in Figure 8, in which A curve represents the transmissivity of PP at different wavelengths before grafting modification (PP) and B curve represents the transmissivity of PP at different wavelengths after grafting modification (TGMPP). It can be seen from the figure that the grafting modification causes the transmissivity of PP to decrease slightly when the wavelength is 980 ± 10 nm.

#### 2.3.3. The Effect of Grafting Modification on the Absorptivity of Polypropylene

**Figure 9:**
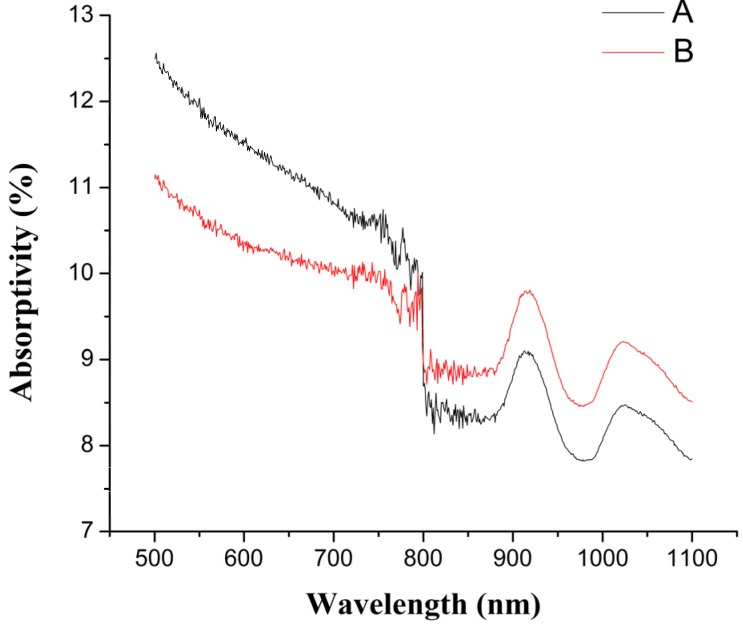
The absorptivity of PP at different wavelength before and after grafting modification.

If the polymers are the upper materials, the high absorptivity of the upper materials will cause only a little laser energy to transmit to the welding zone and a large amount of laser energy will be absorbed by the upper materials, which causes the high processing cost. If the polymers are the lower materials, the high absorptivity of the lower materials will cause a large amount of laser energy to be absorbed by the lower materials, which is good for the laser transmission welding. However, if the absorptivity of the lower materials is too low, the low absorptivity will lead to the low utilization of laser energy and prevent them from being welded with the upper materials under some extreme conditions. So, the effect of grafting modification on the absorptivity of PP needs to be researched. The absorptivity of PP at different wavelengths before and after grafting modification is shown in Figure 9, in which A curve represents the absorptivity of PP at different wavelengths before grafting modification (PP) and B curve represents the absorptivity of PP at different wavelength after grafting modification (TGMPP). It can be seen from the figure that the grafting modification causes the absorptivity of PP to increase slightly when the wavelength is 980 ± 10 nm.

## 3 .The Study of Laser Transmission Welding between TGMPP and PA66

### 3.1. The Preparation of Specimens and Experimental Equipment

To do the next experiments, TGMPP, PP and PA66 are made into the same dimension of L50 mm × W20 mm × H1 mm. Prior to LTW, all samples are cleaned by ultrasonic cleaning machine and then placed in a drying oven for about 12 h. A Compact 130/140 semiconductor continuous laser made by Dilas is used for the experiments. The maximum power of the laser device is 130 W, the output wave-length is 980 ± 10 nm, the minimum beam diameter is 700–800 μm, cooling system is the built-in air cooling system and working temperature is 15–25 °C. The three axis table has a marble pedestal, table stroke is 300 mm × 300 mm × 200mm, repeat positioning accuracy is 0.01 mm and the scope of speed is 0–3000 mm/min.

### 3.2. The Experiments of Laser Transmission Welding between TGMPP and PA66

**Figure 10:**
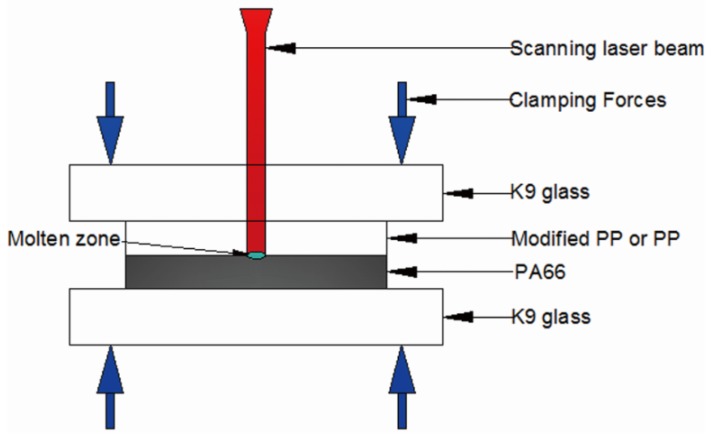
The diagram of laser transmission welding between PP TGMPP and PA66.

In the next experiments, TGMPP and PP are used as the upper materials while PA66 is used as the lower material. Also, the welding zone of PA66 is coated with clearweld as the absorbing layer. As shown in Figure 10, the experiments use K9 glass as the sandwiched layer and adopt lap welding.

Passing through PP or TGMPP, the laser energy is absorbed by the clearweld absorbing layer at the interface of TGMPP or PP and PA66. The laser energy directly acts on PA66. Then, the upper materials (PP or TGMPP) are heated and melted by heat conduction. In the process of solidification, the melted PP or TGMPP and PA66 form the weld seam to connect the upper materials (PP or TGMPP) and the lower materials (PA66).

The evaluations of the laser transmission welding quality are mainly (1) the welding strength; (2) the weld seam width and (3) the appearance of weld seam. In this paper, the welding strength is used to evaluate the quality of the welding.

**Figure 11:**
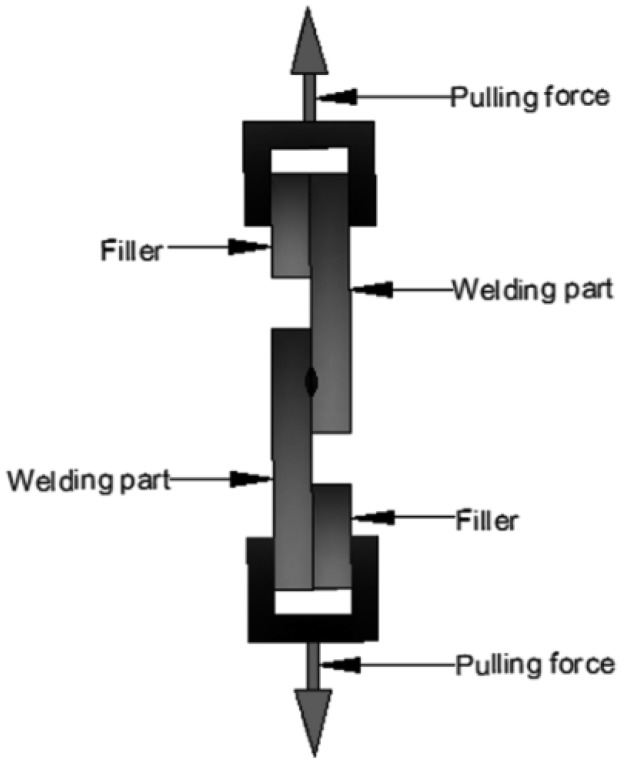
The tensile schematic diagram.

With tensile speed at 2 mm/min and working environment at room temperature, tensile tests finally break the samples (the welding of TGMPP and PA66) through loading tension at both ends of the samples to measure the limit stress of the samples, which can be used to evaluate the welding strength of the samples. The tensile schematic diagram is shown in Figure 11.

The formula used [14] is shown as below.

(2) σ=F/(W×L)

where σ represents the welding strength (MPa); *F* is the maximum pull-off force to make the welding failure (N); *W* is the width of the weld seam (mm) and *L* is the length of the weld seam (mm).

UTM4104 microcomputer control electronic universal testing machine is used to do the tensile tests and a Keyence VHX-1000 ultra-depth electron microscope is used to measure the width of the weld seam.

Through UTM4104 microcomputer control electronic universal testing machine, the maximum pull-off force to make the welding failure can be obtained (*F*)*.* Through Keyence VHX-1000 ultra-depth electron microscope, the width of the weld seam can be obtained (*W*). The length of the weld seam is 20 mm (*L*), which is equal to the width of the original samples (TGMPP, PP and PA66)*.* According to Equation (2), the welding strength can be obtained (σ).

**Figure 12:**
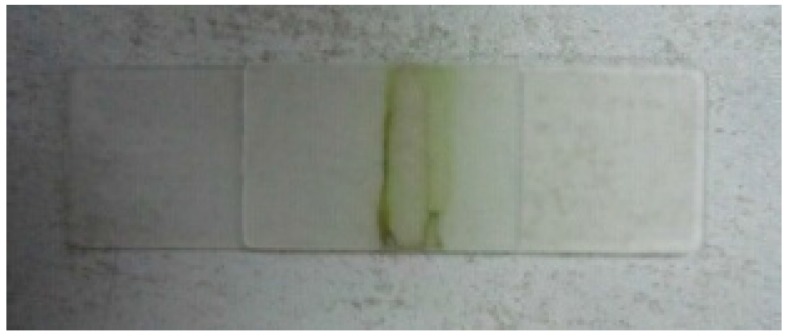
The welding of TGMPP and PA66.

Under the action of laser, only a melted seam on PP can be found and there is no real weld seam between PP and PA66. PP and PA66 cannot be welded together, but TGMPP and PA66 can be welded well. When the laser power is 40 W, the processing speed is 600 mm/min and the clamping force is 0.3 MPa, the welding is shown in Figure 12. Macroscopically, the welding is very good and shapes well, the weld seam is uniform and smooth and no obvious defect is found. Measured by the above method, the welding strength is 3.5 MPa. The strong welding strength proves that the grafting modification can improve the welding performance of PP and PA66 a lot.

### 3.3. Researches on the Mechanism of the Weldability between TGMPP and PA66

#### 3.3.1. Micro Morphology Analysis of the Welding Zone

Firstly, laser is used to weld the upper materials (PP and TGMPP) and the lower materials (PA66). Comparing the micro morphology of the welding zone of TGMPP and PA66 with PP and PA66, TGMPP is researched to investigate how it improves the welding performance of PP and PA66.

Before the observation of the micro morphology of the welding zone between TGMPP and PA66, welding TGMPP and PA66 by laser and putting the welding into formic acid need to be done first. Then, PA66 is dissolved by the formic acid, which makes the micro morphology of the welding zone easy to be observed.

**Figure 13:**
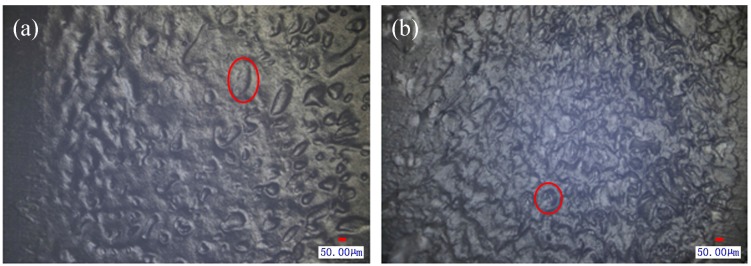
(**a**) The micro morphology of the welding zone between PP and PA66; (**b**) The micro morphology of the welding zone between TGMPP and PA66.

When PP and PA66 are welded by laser, the micro morphology of the weld mark on PP is shown in Figure 13a. Because of the poor compatibility of PA66 and PP, the hot melted plastic molecules cannot diffuse well under the clamping force after they are heated and melted by laser. In microscopic view, the poor welding performance between PP and PA66 is caused by bad adhesion and few holes in the welding zone (as shown in Figure 13a) which makes it difficult to form a good weld seam.

When TGMPP and PA66 are welded by laser, the micro morphology of the weld seam on TGMPP is shown in Figure 13b. In microscopic view, the welding zone of TGMPP has a large number of holes (as shown in Figure 13b). These holes can not only increase the contact area between TGMPP and PA66, but also form mechanical locking structures as similar to the rivet lock to enhance the welding strength greatly. Also, these holes show that the modification of PP can reduce the interfacial tension between PP and PA66, improve their compatibility and their interfacial adhesion and make them easy to diffuse mutually [15]. Because of the mutual diffusion, a large number of locking structures as similar to the rivet lock are formed in the welding zone at last, which improve the welding performance between PP and PA66 and make their welding strength higher.

#### 3.3.2. The Effect of the Bubbles in the Welding Zone on the Welding Quality

In order to investigate the effect of the bubbles caused by high laser power on the welding quality, welding TGMPP and PA66 by laser and putting the welding into formic acid need to be done first. Then, PA66 is dissolved by the formic acid, which makes the micro morphology of the welding zone easy to be observed.

**Figure 14:**
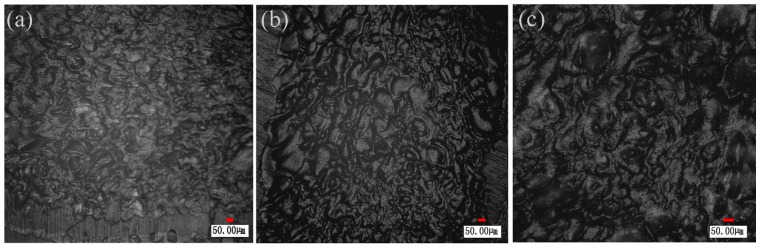
(**a**) The micro morphology of the weld zone on TGMPP when laser power is 20 W; (**b**) 35 W; (**c**) 50 W.

When the processing speed is 10 mm/s, the clamping force is 0.12 MPa and the laser power is 20 W, 35 W and 50 W, respectively, the micro morphologies of the weld zone on TGMPP are shown in Figure 14a–c. It can be found that more and more large holes in the welding zone are formed with the increase of laser power, the reason for which is the large amount of thermal energy caused by high laser power, and the low melting point of TGMPP causes the thermal degradation of TGMPP to form bubbles (holes). These bubbles mainly consist of water vapor, carbon dioxide, carbon monoxide and hydrocarbons [16]. The high pressure generated by these tiny homogeneous bubbles can force upper fused TGMPP to the pits and cracks on the surface of PA66. Thus, the two materials can realize micro-anchor and increase the joining strength. Liu *et al.* studied joining of aluminum alloy to PET using friction lap welding. They also discovered that these bubbles generate high pressure pushing the fused polymers to the pits and micro voids on the surface of metal, which can supply more mechanical bonding and realize tight joining between metal and polymers [17]. Katayama *et al.* also found that evenly distributed bubbles are beneficial to improving the welding strength [18]. Also, it can be concluded that the reasons for the formation of the holes are the improvement of the compatibility between TGMPP and PA66 as mentioned in Section 3.3.1 and the thermal degradation of TGMPP. To some extent, the bubbles are good for the high welding strength. However, when the laser power is too high, the serious thermal degradation causes many large bubbles to be formed in the welding zone as shown in Figure 14c, which account for a wide area in the welding zone and cause the poor welding strength.

#### 3.3.3. X-ray Photoelectron Spectroscopy Analysis of the Welding Zone

To prove that there are chemical reactions in the welding processes, X-ray photoelectron spectrometer (XPS) is used to detect the elements and the chemical bonds in the welding zone of TGMPP after TGMPP and PA66 are welded by laser. The XPS device used in the experiments is Escalab 250Xi manufactured by Thermo Fisher Scientific in Massachusetts, America, which uses the monochromatic Al target as X-ray source.

Before observing the welding zone of TGMPP by XPS, welding TGMPP and PA66 by laser and then putting the welding into formic acid need to be done first. After the formic acid dissolves PA66, TGMPP needs to be washed by formic acid several times and at last by ethanol.

**Figure 15:**
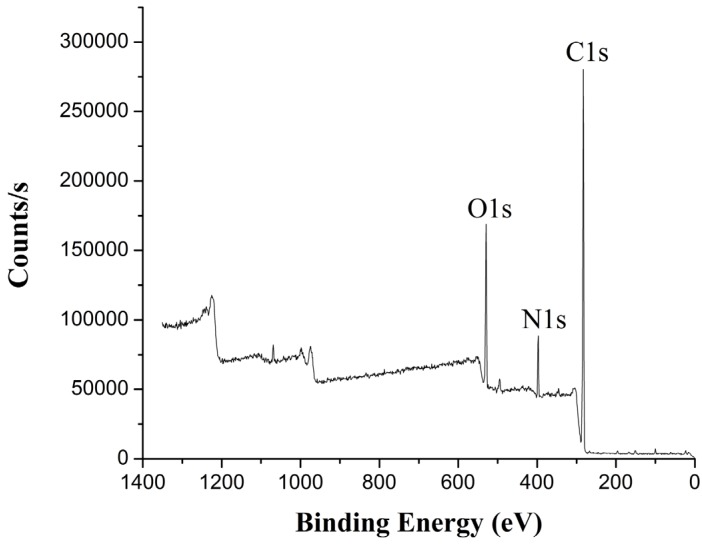
The full element spectrum of the welding zone of TGMPP.

After TGMPP is treated by formic acid and ethanol, the full element spectrum of the welding zone of TGMPP is obtained by XPS as shown in Figure 15. It is found that there are three main elements (carbon, nitrogen and oxygen) in the welding zone. However, there is no nitrogen in TGMPP. So, it is easy to conclude that the nitrogen may come from the reactions between TGMPP and PA66.

**Figure 16:**
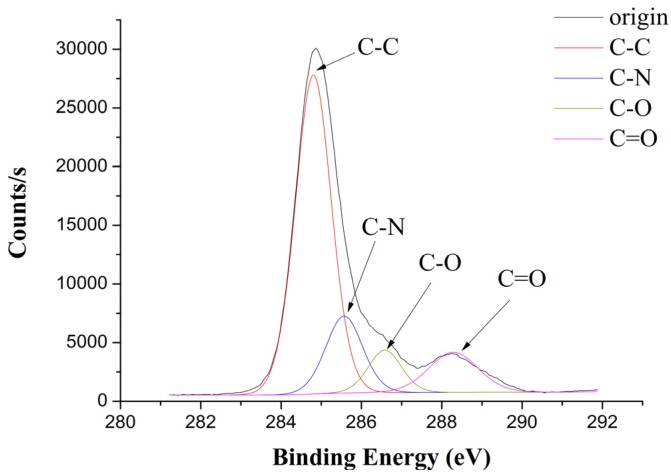
The C1s spectrum of the welding zone of TGMPP.

**Figure 17:**
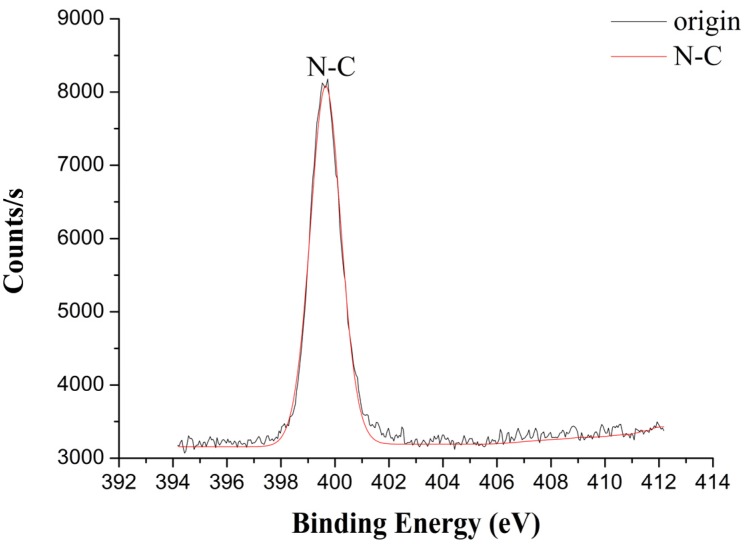
The N1s spectrum of the welding zone of TGMPP.

**Figure 18:**
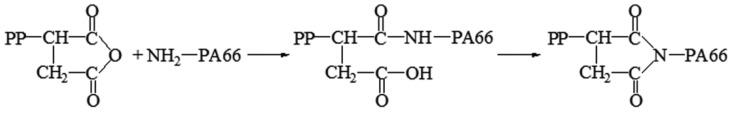
The chemical equations between TGMPP and PA66.

After TGMPP is treated by formic acid and ethanol, the high rate spectrum of the welding zone of TGMPP is obtained by XPS as shown in Figure 16 and Figure 17. The binding energy, FWHM and chemical bonds corresponding to the C1s peak value of TGMPP are shown in Table 1. The binding energy, FWHM and chemical bond corresponding to the N1s peak value of TGMPP are shown in Table 2. From the C1s spectrum (as shown in Figure 16) and Table 1, it is easy to find that in addition to 284.8 eV (C–C), 286.39 eV (C–O) and 287.88eV (C=O), there is a peak at about 285.68 eV, which may belong to the C–N bond. Also, a peak at about 399.73 eV can be found in the N1s spectrum (as shown in Figure 17) and Table 2, which may belong to the N–C bond. However, there are no nitrogen and C–N bonds in TGMPP. So, it is easy to conclude that the nitrogen and C–N bonds are from the reactions between TGMPP and the amino of PA66, and the reactions improve their welding performance dramatically. The chemical equations for PA66 and TGMPP are shown in Figure 18.

**Table 1:** The binding energy, FWHM and chemical bonds corresponding to the C1s peak value of TGMPP.

Binding Energy (eV)	FWHM	Chemical Bonds
284.8	1.11	C–C
285.68	0.96	C–N
286.39	1.03	C–O
287.88	1.26	C=O

**Table 2:** The binding energy, FWHM and chemical bond corresponding to the N1s peak value of TGMPP.

Binding Energy (eV)	FWHM	Chemical Bond
399.73	1.38	N–C

Containing unsaturated double bonds in the molecular structure, MAH is a kind of polar compound with various functional groups, which is easy to react with other polymers under the action of initiator. The strong active anhydride of MAH is easy to react with hydroxyl, carboxyl and amidogen. So, by being grafted to PP, MAH with strong reactive activity can reinforce the affinity and improve the welding performance between PP and PA66.

Gorga *et al.* analyzed the fracture behavior at partially miscible polymer interfaces [19]. Lo *et al.* developed a kinetic model to describe the behavior of semicrystalline polymer interfaces [20]. Horiuchi *et al.* investigated interfacial entanglements between glassy polymers [21]. It can be concluded that when dissimilar polymers are welded, the main mechanism for the improvement of welding strength includes mutual diffusion and molecular entanglement at the interface of the upper materials and the lower materials [19,20,21]. For one thing, the above reactions will reduce interfacial tension between TGMPP and PA66 and increase their compatibility which makes them easy to diffuse mutually. The mutual diffusion cannot only be useful for the formation of the locking holes, but also be good for the formation of blend alloy. For another, because of the chemical reactions, the length of the molecular chain is longer, which is beneficial for the entanglement of the polymer chains. At the same time, researches show that there is a critical molecular weight M_c_, and the entanglement of the polymer chains occurs only when the molecular weight is more than the critical molecular weight M_c_. The chemical reactions can make the molecular weight higher, namely cause the entanglement of the polymer chains to occur easily. Once the entanglement of the polymer chains occurs, the stress of one chain will spread to other molecular chains, which improves the welding strength. Furthermore, the chemical reactions form C–N bonds between the chains of TGMPP and PA66, which are also good for the improvement of the welding strength.

## 4. Conclusions

In this paper, using MAH to improve the welding performance of PP and PA66 by being grafted to the side chain of PP and the welding mechanism based on modification of PP for improving the laser transmission weldability to PA66 are researched. The following conclusions can be drawn from the experiments in this paper.

(1) Through grafting reactions, MAH with strong reactivity and polarity can be grafted to the side chain of PP to improve the welding performance of PP and PA66.
(2) In general, the grafting modification has certain influence on the mechanical and thermal properties of PP. However, the original properties of PP change little.
(3) The grafting modification has little influence on the optical properties of PP. The grafting modification causes the reflectivity and transmissivity of PP to decrease slightly and makes the absorptivity of PP increase slightly.
(4) TGMPP and PA66 can be welded well. The micro morphology of the welding zone displays that because of the grafting modification, the affinity and the compatibility between PP and PA66 are improved and a large number of locking structures as similar to the rivet lock are formed, which is one of the reasons why the welding performance is so high.
(5) Proved by the X-ray photoelectron spectroscopy (XPS), PA66 may react with the MAH of the side chain of TGMPP under the heating effect of laser, which is another reason for the high welding performance.
(6) The evenly distributed bubbles caused by the thermal degradation are good for the high welding strength. However, when the laser power is too high, the serious thermal degradation causes many large bubbles to be formed in the welding zone, which account for a wide area in the welding zone and cause the poor welding strength.
